# Narrative Matters: Self‐harm in Britain post‐1945: the evolution of new diagnostic category

**DOI:** 10.1111/camh.12227

**Published:** 2017-07-06

**Authors:** Chris Millard, Dennis Ougrin

**Affiliations:** ^1^ University of Sheffield Jessop West, 1 Upper Hanover Street Sheffield S3 7RA UK; ^2^ Child and Adolescent Psychiatry Institute of Psychiatry, Psychology, and Neuroscience Kings College London London UK

Self‐harm (SH) is a common problem in adolescent and young adult mental healthcare, with an extensive literature addressing the issue. However, there is still much confusion about what self‐harm involves, and one recent article suggests that even defining self‐harm ‘continues to be a challenge’ (Chandler, King, Burton, & Platt, 2015). This article offers an overview of the history and the evolution of the concept identifying a range of factors that have shaped its course to date.

‘Self‐harm’ can be defined as self‐injury or self‐poisoning, irrespective of the intent of the action. It used to be referred to as ‘deliberate self‐harm’ (DSH) before a representation from service users objecting to the pejorative flavour of the term ‘deliberate’ resulted in its removal (i.e. ‘DSH’ to ‘SH’). Self‐harm encompasses a wide spectrum of behaviours that can be categorised into self‐harm with suicidal intent (suicide attempts), self‐harm without suicidal intent and self‐harm with undetermined intent.

It was Kreitman's seminal work on parasuicide that was presented in the British Medical Journal in 1969 that led to the broadly defined concepts underpinning contemporary definitions of suicidal behaviour used across Europe today (Hjelmeland et al., 2002). Kreitman's broadly defined concepts contrasted with the contemporary American perspective based on the work of Beck, Beck, & Kovacs (1975), which viewed intent as key to the classification of suicidal behaviour. This historical divide between those who believed that self‐harm should be categorised on the basis of intent to die, and those who believe that self‐harm should represent a broad spectrum of self‐harmful behaviour irrespective of suicidal intent, remains to this day. In addition, the term nonsuicidal self‐injury, defined as the direct, deliberate destruction of one's own body tissue in the absence of intent to die has gained prominence of late, especially in the United States, and has been included in DSM 5 as a condition for further study. In the United Kingdom, however, the National Institute for Health and Care Excellence (NICE) continues to define self‐harm as self‐poisoning or self‐injury, irrespective of the apparent purpose of the act.

Suicide remains the second leading cause of death in adolescents, and self‐harm remains the best predictor of death by suicide. There has been a significant increase in the number of young people presenting with serious self‐harm to Accident and Emergency (A&E) departments in recent years, from 13,054 in 2005/2006 to 19,642 in 2014/2015 in England alone (NHSDigital, 2016). The number of girls treated after cutting themselves has almost quadrupled over the same period, from 600 to 2311 – a 285% rise. The number of girls whom A&E teams have treated after attempted hanging has risen during that decade, from 29 to 125. While far fewer boys present to A&E after cutting themselves, the numbers went up from 160 in 2005–06 to 457 in 2014–15 – a rise of 186%. Similarly, the numbers of boys who attempted hanging also doubled from 47 to 95 over the same period.

The history of SH in Britain since 1945 roughly falls into two periods: 1945–1980, and 1980‐present. In the first period, self‐harm is characterised (broadly) to involve overdosing, which is perceived to be a cry for help; in the second period, it is more often seen to involve self‐cutting or self‐burning in order to regulate intolerable tension, or feelings of emotional numbness. In both periods, SH is viewed as being a problem affecting predominantly young people (i.e. late teenagers to c.30 years old). The specific concern over children under 18 self‐harming is very new indeed, and there exist almost no studies before the 1990s.

The history of self‐harm by self‐injury and by self‐poisoning is also instructive. The concern with self‐cutting, especially among young White females (Steggals) ‘genuinely demographically dominant, teenage White female’) principally emerged from American psychiatric inpatient units. A path‐breaking clinical study in 1960 and an orthodox sociological analysis in 1964 brought to light an ‘adolescent scarification crisis’ in a prestigious Chicago psychiatric hospital. In the United Kingdom, an unpublished study at the Maudsley on ‘self‐inflicted injuries’, was also centrally concerned with self‐cutting (McEvedy, 1963). Concern about this kind of psychiatric inpatient behaviour was given a large boost in visibility through a number of studies in the late 1960s and early 1970s in both Britain and the United States. These formed the roots of the current view of SH‐as‐self‐cutting, and were solidified through Favazza's influential book *Bodies under Seige* (1987).

Self‐poisoning, by contrast, emerged in Britain from psychiatric observation wards (secure spaces connected to general hospitals, usually former workhouse ‘mental blocks’) or accident and emergency departments of general hospitals. Batchelor (1955) and Stengel, Cook, and Kreeger (1958) conducted landmark studies in the 1950s, taking advantage of these unusually close ties between psychiatry and general hospitals. As a result, an increasing number of self‐poisoning patients were subjected to psychiatric assessment, and described as making an appeal to a social circle, a ‘cry for help’, rather than trying (and failing) to kill themselves. This socially focused assessment was aided by the development of psychiatric social work, and chimed with the interpersonal, social outlook of the psychiatry of that period.

Self‐poisoning became a national concern in 1960s in Britain. The Mental Health Act 1959 removed all legal restrictions to treating mental illness in general hospitals, and the Suicide Act 1961 decriminalised attempted suicide, taking police action out of the equation. However, there was still a sense that some form of medical intervention was required, and very soon after the Suicide Act came into effect, the Ministry of Health issued a memorandum to hospitals recommending that all ‘attempted suicides’ that present at casualty be subject to psychiatric assessment. Thus on a national scale, self‐poisoned patients began to be investigated by psychiatrists, psychologists and psychiatric social workers, creating nationwide visibility for this seemingly new problem.

In addition, a number of ‘social and cultural’ factors also contributed to the way we perceive self‐poisoning and self‐injury. Concerns over prescription medicine in the 1960s (most obviously and tragically in the Thalidomide disaster) were widespread; a number of campaigns urged the disposal of dangerous unused prescription medicines at home; self‐cutting achieved prominence – in part – because of its inclusion in the new Diagnostic and Statistical Manual of Mental Disorders (DSM‐III) (1980), as a symptom of borderline personality disorder.

However, blanket terms like ‘social’ and ‘cultural’ do not really clarify what is happening when new behaviours come to prominence. Practice‐based ‘methodological’ issues are crucial. For example, it was a result of the employment of psychiatric social workers that the idea of a ‘social context’ is brought to bear on presentations of overdosing.

The normal reaction upon discovering somebody who has engaged in self‐cutting of the forearms or wrists is likely to involve first aid that can be given by the nonmedically trained, followed by a trip to the GP for possible referral for psychotherapy. It is relatively unlikely to end up at A&E, unless the wrists have been cut, or there is an unusual amount of blood. Contrast this with discovering someone who has taken an uncertain number of pills, even a relatively small amount. People are much more likely to call an ambulance or go to A&E because they cannot gauge the severity of the danger as easily, or know the drug's effects, and therefore this form of SH is automatically medicalised. Thus, self‐cutting cases largely end up in studies that use counselling; self‐poisoning cases are presented in epidemiological studies issuing from general hospitals. Research, however, does not support the notion of self‐injury as low‐risk behaviour. NSSI is strongly associated with suicide attempts, and the two behaviours often co‐occur, with an increase in one behaviour associated with an increase in the other. In fact, in depressed adolescents, a history of NSSI behaviour may be an even stronger predictor of future suicide attempts than recent suicide attempts. In addition, young people with self‐harm by self‐cutting are more likely to die of suicide than those with self‐poisoning (Hawton et al., 2012).

In summary, the history of self‐harm research is complex and blighted by inconsistencies in its definition. The main disagreement among researchers lies in the role of suicidal intent in the definition of self‐harm. In the United Kingdom, self‐harm is most commonly defined as a broad spectrum of behaviours. People with suicidal and nonsuicidal self‐harm may, however, differ in important ways and research into specific self‐harm behaviours is required to establish diagnostic validity of self‐harm subtypes which can allow services to tailor treatment.
